# Impact of dental loupes and operating microscopes on restorative performance of preclinical and clinical dental students: a pilot study

**DOI:** 10.1186/s12909-026-08904-6

**Published:** 2026-02-26

**Authors:** Elisabeth Prause, Jonas Rechlin, Simon Peroz, Anna Steinke, Manja von Stein-Lausnitz, Florian Beuer

**Affiliations:** Department of Prosthodontics, Geriatric Dentistry and Craniomandibular Disorders, Charité-Universitätsmedizin Berlin, corporate member of Freie Universität Berlin and Humboldt-Universität zu Berlin, Aßmannshauser Str. 4-6, Berlin, 14197 Germany

**Keywords:** Magnification, Education, Dentistry, Dental loupes, Dental operating microscope, Restorative dentistry

## Abstract

**Background:**

The integration of magnification devices into dental education is widely recommended, yet evidence regarding their impact on student performance remains limited. The present study evaluated the effect of dental loupes (DL) and dental operating microscopes (OPMI) on the quality of crown preparation and provisional restoration among preclinical and clinical dental students compared with standard protective glasses (SG).

**Methods:**

Sixty dental students participated: 30 preclinical (fifth semester) and 30 clinical (ninth semester). Participants were randomly assigned to three groups: DL (ZEISS EyeMag Pro S 3.5 × 400), OPMI (ZEISS EXTARO 300), or SG (control). Each prepared a crown and a provisional restoration. Outcomes were assessed conventionally (expert ratings) and digitally (objective parameters). Performance differences were analyzed using generalized estimating equations (*p* < 0.05).

**Results:**

Optical magnification significantly improved restorative performance compared with SG. Preclinical students using OPMI achieved the highest overall scores, while clinical students performed best with DL. The Generalized Estimating Equations (GEE) confirmed significant differences between OPMI and SG, but not between preclinical and clinical groups.

**Conclusions:**

Optical magnification improved restorative outcomes compared with standard glasses. OPMI use was most beneficial in preclinical training, whereas DL appeared more advantageous in clinical settings. Integrating optical magnification into dental education enhances skill acquisition and clinical quality. Within the limitations of the present pilot study, microscope-assisted training improved the quality of restorative preparation outcomes in a preclinical student setting. Further studies are required to confirm these findings in larger cohorts and clinical environments.

## Background

In dentistry, the ability to perform precise tooth preparations is crucial for successful restorative treatments [1]. The use of magnification devices such as dental loupes (DL) and dental operating microscopes (OPMI) has gained increasing attention in dental education due to their potential to enhance the visualization of morphological details, ultimately leading to improved preparation quality [1, 2]. While some studies suggest that loupes contribute to better preparation accuracy, others have found no significant effect on the quality of full-coverage crown preparations or on the visual acuity of dental students [1, 3, 4]. Another study revealed that the application of higher magnification during tooth preparation significantly influenced the extent of marginal discrepancies in computer-aided design (CAD)/computer-aided manufacturing (CAM) crowns [5]. Preparations performed with fine-grit diamond rotary instruments under microscopic magnification, exceeding that provided by loupes, resulted in superior marginal adaptation characterized by smaller gap dimensions [5].

Minimally invasive preparation techniques have become the standard of care, as preserving tooth structure directly influences treatment adherence and the longevity of restorations. The use of modern optical magnification devices, in combination with appropriate lighting systems, may contribute to achieving these conditions [6, 7]. DL have been established for many year and studies have been widely published regarding advantages of DL [6, 8–12]. Contrary, literature about OPMI is reduced. It is known that OPMI offer the highest magnification degree of all magnification devices and the most neutral working posture [13, 14] and it is used for endodontic treatment procedures [15, 16]. Furthermore, dentist´s posture is improved and ergonomic benefits are proven [13, 14]. Studies showed that both magnification devices had a positive impact on dental treatment procedure [13]. So far, no data is available regarding the use of OPMI in dental education for restorative treatment procedures.

Interestingly, while magnification is widely recognized for its benefits, its implementation in dental education remains inconsistent. Nearly one-quarter of all magnification users have indicated that they are unlikely to use magnification in the teaching environment, even though they may rely on it in private practice [17]. Several explanations have been given for the mentioned discrepancy, including institutional resistance and personal preference [17]. Therefore, only a small amount of institutions supports its introduction in the first year of the curriculum, even though it could be proven that magnification positively impacted undergraduates dental students´ performance in cavity preparations [1, 9]. Furthermore, improvements could be shown regarding posture, hand skills and procedure quality [13, 18]. These findings suggest that educational institutions should address faculty concerns and resistance before mandating the use of magnification devices in dental training programs.

However, the implementation of magnification devices in the curriculum can be used at any stage of clinical experience, regardless of professional skill level [6]. Adequate training is recommended to ensure proper utilization and maximize its benefits [6, 19]. Magnification devices offer significant advantages by improving visual perception, enhancing hand-eye coordination and facilitating spatial awareness, thereby allowing for more detailed observation of dental cavities [4, 6].

Improved magnification ensures greater precision in workflow and provides better visibility during restorative treatments [1, 9]. This aspect, in turn, facilitates the detection of restoration margins regarding restorative dentistry. Furthermore, the removal of excess composite resin, and more precise finishing of preparation edges, ultimately minimizing unnecessary tooth structure removal would be advantageous [20, 21]. However, despite these potential benefits, further research is needed to assess the long-term effects of OPMI on both the learning process of dental students and the overall quality of dental treatments.

While the benefits of dental loupes have been widely discussed, evidence regarding the educational impact of OPMI in restorative undergraduate training remains limited. In particular, comparative data evaluating loupes, microscopes, and standard visualization within the same standardized educational framework are scarce. Therefore, the present pilot study was designed as hypothesis-generating feasibility research. The present pilot study aimed to explore the association between different visualization conditions (standard protective glasses [SG], DL, and dental OPMI) and restorative performance outcomes under standardized simulation conditions on the preparation quality and working techniques of preclinical and clinical dental students. The null hypothesis was that there would be no significant difference in preparation quality between OPMI, DL, and SG.

## Methods

The present clinical study was approved by the local Ethics Committee in 2020 (application number: EA1/250/23). Since then, it is being conducted in accordance with the Declaration of Helsinki on Ethical Principles for Medical Research. The study was designed as a controlled, randomized, between-group pilot study conducted under standardized preclinical simulation conditions.

Sixty dental students were recruited for the preparation exercise. The cohort consisted of 30 students from the 5th preclinical semester and 30 students from the 9th clinical semester. The average age of the study participants was 24.7 years. A total of 20 male and 40 female students participated. On average, students in the 9th semester were two years older than those in the 5th semester. However, differences in baseline manual dexterity and clinical experience between cohorts must be considered when interpreting the results.

At baseline, participants were asked about their previous experience with magnification aids (e.g., dental loupes and/or operating microscope) as well as prior participation in similar preparation exercises. As part of the undergraduate curriculum, students of both cohorts had no routine clinical experience with dental operating microscopes for restorative procedures. Therefore, prior OPMI experience was neither expected nor used as an exclusion criterion.

Participants were recruited via flyers and seminar presentations and randomly assigned to three groups (SG, DL or OPMI). Randomization was performed separately within each cohort (fifth and ninth semester) to ensure comparable distribution of participants. Therefore, a group allocation was performed by simple randomization using sealed envelopes. Group allocation was implemented before the start of the preparation exercise, and participants received device-specific instructions according to their assigned group. Due to the nature of the intervention (use of optical magnification devices), blinding of raters to the assigned visualization condition was not feasible.

All participants received oral and written information about the study, with sufficient time to give informed consent; participation required a signed consent form, and data were pseudonymized.

Groups differed by magnification device: [1] protective eyewear without magnification (Virtua safety glasses, 3 M) [2], dental loupes with headlamp (ZEISS EyeMag Pro S 3.5×), and [3] dental operating microscope (ZEISS EXTARO 300). Each group received an introductory session on their device. Loupes had fixed magnification (3.5×, 40 cm working distance), while microscopes allowed adjustable magnification; interpupillary distance and viewing angle were adjusted for ergonomic posture.

The task was preparation of a right maxillary central incisor for an unveneered lithium disilicate crown and fabrication of a provisional restoration (Luxatemp Auto Plus, DMG) within 150 min. Preparation followed preclinical standards, with workflow and remaining time displayed on a screen. Models, provisional restorations, and silicone keys were collected, and teeth were scanned (Primescan, Dentsply Sirona). An anterior tooth was selected to ensure standardization and optimal comparability of preparations. The accessibility of anterior teeth may also facilitate microscope positioning.

All preparations were performed under standardized preclinical simulation conditions on dental training models mounted in phantom heads. The phantom units (DSEclinical type 5198; KaVo, Biberach, Germany) were prepared for the present study and equipped with face masks (DPS; KaVo, Biberach, Germany) and training models (ANA-4; Frasaco, Tettnang, Germany). The models were fixed to a training workstation and positioned to replicate a typical clinical working situation. The same phantom head setup, tooth position, and working conditions were used for all participants to ensure comparability across study groups.

Evaluation was analog and digital. The combination of conventional and digital assessments was intentionally chosen to reflect contemporary educational evaluation strategies rather than to determine superiority of one assessment method. Analog assessment used a dental model and periodontal probe (PA probe/UNC15, HuFriedy Group) by three raters (10th-semester dental student, dentist with 1 year of clinical experience, and senior clinician/attending dentist with 7 years of clinical experience) under natural light, based on preclinical scoring sheets (Tables 1 and 2).

**Table 1:** Analog evaluation criteria for tooth preparation and provisional restoration based on standardized preclinical assessment rubrics. Each parameter was scored on a 0–3 scale by three independent raters (student, dentist, senior clinician)

**Category**	**Score**			
	**3**	**2**	**1**	**0**
Circumferential reduction	Reduction meets the guidelines (1–1.2 mm)	Minor correctable deviations	Major correctable deviations	Circumferential reduction does not meet the guidelines
Tooth shape	Correct tooth shape, cusp beveling and crown inclination considered	Minor correctable deviations	Major correctable deviations	Incorrect tooth shape, cusp beveling, and/or crown inclination not considered
Occlusal reduction	Occlusal reduction meets the guidelines	Insufficient substance removal (1.1–1.5.1.5 mm)	Excessive substance removal (2.0–2.5.0.5 mm)	More than 3 mm or less than 1 mm occlusal reduction
Preparation margin – smoothness	Chamfer is smooth and polished	Chamfer with minor correctable irregularities	Chamfer with major correctable irregularieties	Incorrect preparation margin (shoulder/tangential), non-correctable irregularties
Preparation margin – height	Uniformly 0.5 mm supragingival to epigingival	0.1–0.4.1.4 mm subgingival or 0.6–0.9.6.9 mm supragingival	0.5 mm subgingival or more than 1 mm supragingival	0.6–0.9.6.9 mm subgingival
Taper Angle	6°, no undercuts	4°−5°, 7°−8°, not undercutting	<4°, 9°−10°, not undercutting	>10° or undercutting
Path of insertion	Path of insertion maintained	Minor correctable deviations	Major correctable deviations	Path of insertion not maintained, non-correctable

**Table 2:** Conventional evaluation criteria applied for assessment of provisional restorations under standardized preclinical conditions. All items were rated on a four-point ordinal scale (0–3)

**Category**	**Score**			
	**3**	**2**	**1**	**0**
Impression for provisional restoration	Adequate tooth reproduction, correctly trimmed and dimensioned, no distortions in relevant areas	Minor correctable deviations	Major correctable deviations	Distortions affecting prepared teeth, tooth not fully/incorrectly reproduced, catalyst residues
Hygiene suitability of the provisional restoration	Margin not overcontoured, interdental cleaning possible	Margin slightly overcontoured, interdental cleaning limited	Margin markedly overcontoured, interdental cleaning severely limited	Margin irregular and overcontoured, interdental cleaning not possible
Marginal fit of the provisional restoration	Clean circumferential marginal fit, gap < 0.1 mm	Marginal gap in isolated areas, gap < 0.5 mm	Marginal gap in multiple areas, gap < 1.0 mm	Circumferential marginal gap, poor fit and > 1.0 mm
Proximal contacts	Both proximal contacts present and not too tight	Proximal contacts too tight	Only one proximal contact present	Both proximal contacts missing
Polishing of the provisional restoration	Clean high-gloss polish	Clean polish, but no high-gloss finish	Only roughly finished, no polishing	Poor finishing, no polishing

Digital assessment compared scans to a master model using prepCheck software, generating color-coded deviation maps for scoring. Scores (0–3) were identical for both methods; provisional restorations were assessed only conventionally. Five assessments were performed: analog provisional, analog preparation, digital preparation, combined preparation, and overall assessment. Age and gender were recorded descriptively. Working hours, fatigue-related parameters, and visual strain were not systematically assessed.

To perform statistical analyses, SPSS 28.0 (IBM) and the online tool DataTab (DATAtab e.U.) was used. Statistical significance was set as *p* < 0.05 probability level. Inter-rater agreement was evaluated using the intraclass correlation coefficient (ICC), and Bland-Altman plots assessed method agreement. Differences between semesters and magnification aids were analyzed using Generalized Estimating Equations (GEE), accounting for rater clustering. All outcomes were reported as percentages of the maximum achievable score to allow comparability across items and scoring domains.

## Results

Results should be interpreted cautiously due to the exploratory nature and pilot sample size. The analysis of average percentage scores relative to maximum achievable points revealed distinct performance patterns depending on the magnification aid (Table 3.). When comparing student cohorts from different semesters, students using the OPMI achieved the highest mean scores in preparation assessments, 4.68% in conventional and 69.13% in digital evaluations, followed by the DL group (70.08% and 64.68%, respectively) (Table 3.). The SG group consistently showed the lowest results (68.02% conventional, 60.00% digital). Similar trends were found in overall evaluations, where DL users reached the highest mean score (65.63%), followed by OPMI (63.32%) and SG (59.64%) (Table 3.). In the evaluation of provisional restorations, ninth-semester students using DL achieved the best results (≈ 60%), while fifth-semester students with OPMI scored lowest (36.54%) (Table 3.).

**Table 3:** Mean percentage scores relative to the maximum achievable points across evaluation methods, stratified by semester and visualization group (SG, DL, OPMI). Values represent mean ratings of three independent evaluators (student, dentist, senior clinician)

Semester	Group	n	Mean percentage scores relative to the maximum possible points across evaluation methods														
			Provisional restoration			Preparation conventional			Preparation digital			Preparation conventional + digital			Overall assesment		
			ST	D	SC	ST	D	SC	ST	D	SC	ST	D	SC	ST	D	SC
			Æ			Æ			Æ			Æ			Æ		
Fifth Semester	SG	10	30.4	55.6	48.9	55.7	76.2	70.9	70.9	55.7	58.6	63.6	66.0	64.9	55.9	64.4	62.2
			44.9			67.6			61.8			64.8			60.9		
	DL	10	31.7	55.8	40.0	55.2	64.3	69.0	69.0	48.1	54.8	64.0	63.1	63.8	59.4	65.2	60.0
			42.5			66.2			57.3			63.6			61.5		
	OPMI	10	22.9	50.4	36.3	65.7	85.7	76.2	74.3	77.1	65.7	71.1	81.8	72.0	59.1	73.1	62.8
			36.5			75.9			72.4			75.0			65.0		
	Total	30	28.2	53.8	41.8	58.9	78.3	72.1	71.4	60.3	59.7	66.2	70.3	66.9	58.1	67.7	61.7
			41.3			70.0			63.8			67.8			62.5		
Ninth Semester	SG	10	52.5	69.2	69.2	57.6	78.1	69.5	71.9	39.5	63.3	63.6	58.0	65.1	57.9	57.9	58.9
			56.9			68.4			58.3			62.2			58.3		
	DL	10	55.0	70.0	55.8	63.8	83.3	74.8	85.2	65.7	65.2	74.2	74.2	70.9	68.9	73.3	66.9
			60.3			74.0			72.1			72.8			69.7		
	OPMI	10	41.9	60.0	51.4	70.0	75.7	74.8	67.1	68.1	62.4	68.4	71.6	68.4	61.7	69.3	63.6
			51.1			73.5			65.9			69.5			64.8		
	Total	30	50.1	66.7	52.2	63.8	79.0	73.0	74.8	57.8	63.7	68.7	67.9	67.9	62.9	66.7	63.1
			56.3			72.0			65.4			68.2			64.3		
Total	SG	20	40.8	61.9	49.0	56.7	77.1	70.2	71.4	47.6	60.9	63.6	62.0	65.0	56.9	61.4	60.7
			50.6			68.0			60.0			63.5			59.6		
	DL	20	43.3	62.9	47.9	59.5	78.8	71.9	77.1	56.9	60.0	69.1	68.7	66.9	64.2	69.3	63.4
			51.4			70.1			64,.8			68.2			65.6		
	OPMI	20	31.2	54.6	42.9	67.9	80.7	75.5	70.7	72.6	64.0	69.8	76.7	70.2	60.2	71.5	63.1
			42.9			74.7			69.1			72.2			64.9		
	Total	60	38.5	59.9	46.7	61.3	78.9	72.5	73.1	59.0	61.7	67.5	69.1	67.4	60.3	67.2	62.4
			48.3			70.9			64.6			68.0			63.3		

*ST* Student, *D* Dentist, *SC* Senior clinician, *n* number of cases, *Æ* mean values of all three raters

Comparing semesters, fifth-semester students performed best with OPMI, particularly in preparation and overall evaluations, whereas ninth-semester students using DL slightly outperformed the OPMI group in digital and combined assessments. Across all measures, SG use was associated with the lowest performance (Table 3.). Overall, both OPMI and DL contributed to superior results, while reliance on SG limited performance.

**Fig. 1:**
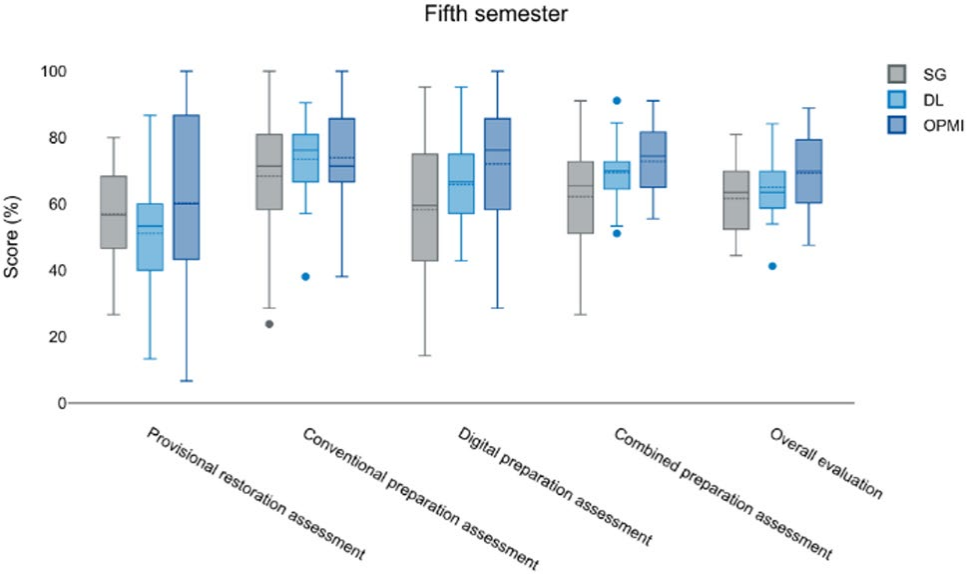
Representation of the results from various assessments conducted during the fifth semester, measured in percentage scores. The evaluated categories include the Provisional Restoration Assessment, the Conventional Preparation Assessment, the Digital Preparation Assessment, the Combined Preparation Assessment, and the Overall Evaluation. The three performance groups of students, SG (gray), DL (light blue), and OPMI (dark blue) are displayed

In the fifth semester, OPMI users achieved the highest conventional (75.9%) and digital (72.4%) preparation scores, resulting in a combined mean of 65.0% (Fig. 1).

**Fig. 2:**
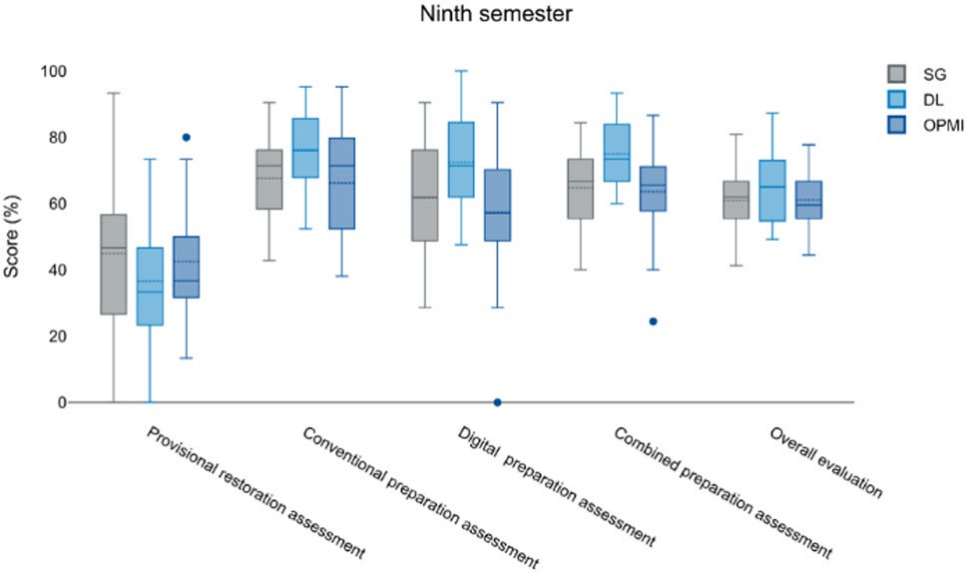
Representation of the results from various assessments conducted during the ninth semester, measured in percentage scores. The evaluated categories include the provisional restoration assessment, the conventional preparation assessment, the digital preparation assessment, the combined preparation assessment, and the overall evaluation. The three assessed groups, SG (gray), DL (light blue), and OPMI (dark blue), display different performance levels

In contrast, DL users achieved higher scores with the highest performance observed in the ninth semester (73.9% under conventional evaluation and 72.8% under digital evaluation), corresponding to a combined mean score of 69.7% (Fig. 2).

Due to the clustered data structure (three evaluations per work), Generalized Estimating Equations (GEE) were applied to account for within-cluster correlations. The GEE analysis revealed significant differences between magnification aids. In the fifth semester, the OPMI group performed significantly better than the SG group in conventional (*p* = 0.006), digital (*p* = 0.028), and combined preparation assessments (*p* = 0.003). Additionally, the DL and OPMI groups differed significantly in digital preparation (*p* = 0.004). In the ninth semester, significant differences were found between SG and DL in digital (*p* = 0.015) and combined (*p* = 0.040) preparation. Across all semesters, a significant difference was observed between SG and OPMI in digital preparation (*p* = 0.018). No significant differences emerged between semesters when comparing overall performance (*p* < 0.05).

**Fig. 3:**
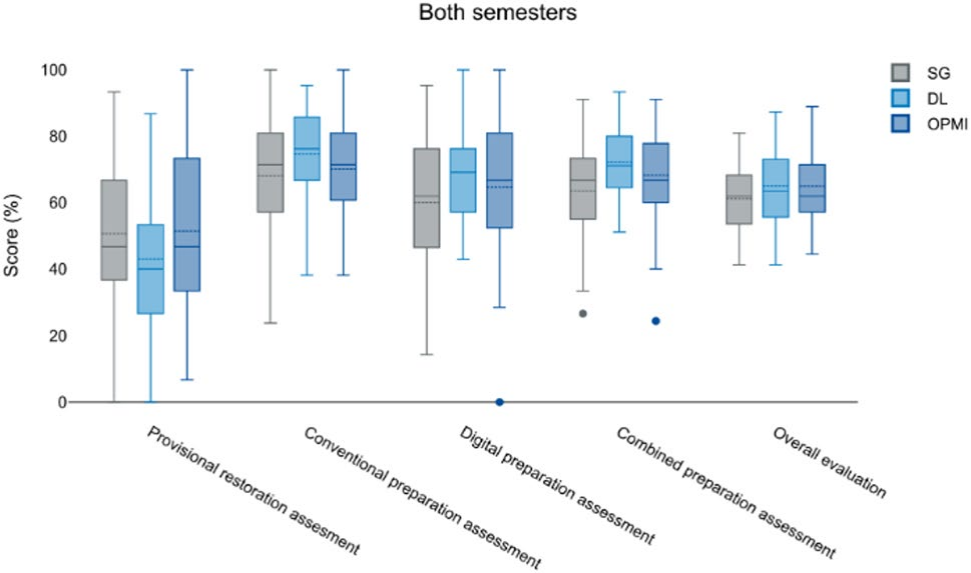
Comparison between the 5th and 9th semester regarding the different evaluated parameters (provisional restoration, conventional and digital preparation, combined preparation assessment and overall evaluation). The different study groups (DL and OPMI) and the control group (SG) were evaluated. There were only significant differences with respect to the digital preparation assessment (OPMI) and the combined preparation assessment (SG and OPMI)

When all data were pooled, the DL group achieved the highest preparation averages (70.9% conventional; 64.6% digital; 72.2% combined) and the best overall results (65.6%), followed by OPMI (63.3%), while SG consistently ranked lowest (Table 3.; Fig. 3).

Due to the clustered data structure (three evaluations per work), Generalized Estimating Equations (GEE) were applied to account for within-cluster correlations. The GEE analysis revealed significant differences between magnification aids. In the fifth semester, the OPMI group performed significantly better than the SG group in conventional (*p* = 0.006), digital (*p* = 0.028), and combined preparation assessments (*p* = 0.003). Additionally, the DL and OPMI groups differed significantly in digital preparation (*p* = 0.004). In the ninth semester, significant differences were found between SG and DL in digital (*p* = 0.015) and combined (*p* = 0.040) preparation. Across all semesters, a significant difference was observed between SG and OPMI in digital preparation (*p* = 0.018). No significant differences emerged between semesters when comparing overall performance (*p* < 0.05).

Interrater reliability analysis using the Intraclass Correlation Coefficient (ICC) showed the highest agreement for conventional preparation (ICC = 0.827), followed by provisional restoration (ICC = 0.779) and overall evaluation (ICC = 0.771). The lowest reliability occurred in the digital preparation evaluation (ICC = 0.615), indicating greater variability among evaluators. All ICC values were statistically significant (*p* < 0.001).

**Fig. 4:**
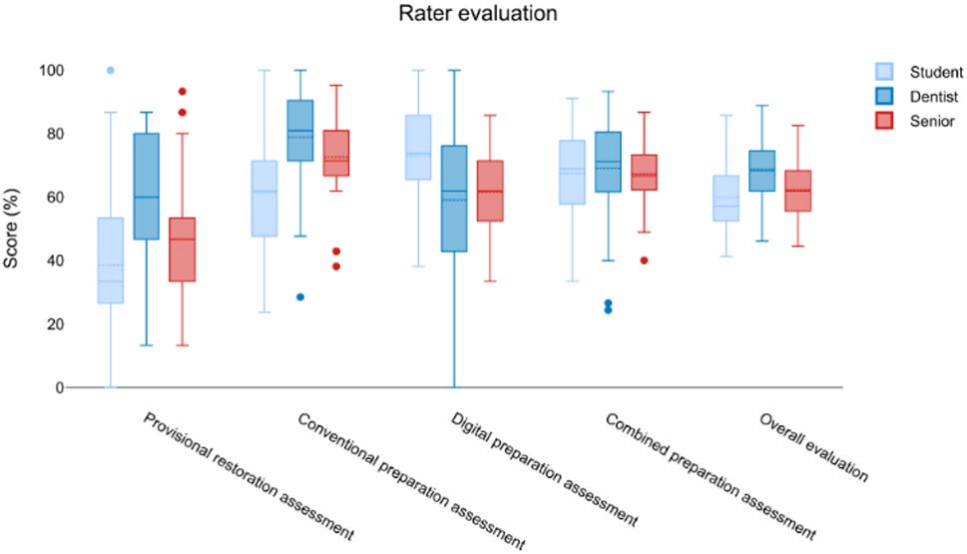
Rater evaluation regarding the different parameters (provisional restoration, conventional and digital preparation, combines preparation assessment and overall evaluation). Students´ evaluation is shown in light blue, dentists´ evaluation is depicted in dark blue whereas the senior clinicans´ evaluation is represented in red. There was a general high agreement for all evaluated parameters between the raters despite their different level of experience

In summary, OPMI and DL use were associated with superior performance in both preparation and overall assessments, while SG resulted in consistently lower outcomes. Interrater agreement was highest in conventional and provisional restoration evaluations, and lowest for digital assessments (Fig. 4).

## Discussion

The present pilot study aimed to evaluate the impact of magnification devices on preparation quality and working techniques of dental students. The null hypothesis was partially rejected, as significant differences were observed between visualization conditions in several evaluation domains. The results of the present study indicate that the use of magnification aids generally improved evaluation outcomes, with variations depending on semester and assessment method. Ninth-semester students consistently achieved higher scores than those in the fifth semester. The three evaluators (student, dentist, senior clinician) showed differing assessment tendencies, with the greatest variability observed in digital preparation evaluations. Correlations between age, working hours, and outcomes were mostly nonsignificant, and gender had no effect. The general performance improvement observed with magnification devices aligns with earlier findings [8, 9, 22]. Studies have shown that DL enhances psychomotor skills, reduces task time, and improves performance quality, although some reported no statistically significant differences between groups [8, 12, 23–25].

In line with prior work, preclinical students in the present study showed improved performance with OPMI, possibly due to higher motivation and novelty of the technique. Clinical students performed better overall, likely owing to existing practical skills. Thus, magnification aids improved performance in general, though not with statistically significant skill progression, and no clear preference emerged between OPMI and DL. No correlation between working time, age, and preparation results was observed.

Both digital and analogue evaluations were applied, assessed by three raters with different experience levels. High interrater consistency was found, and no significant differences between digital and conventional assessments were detected. Hence, neither evaluation method proved superior. Previous studies have shown that conventional assessments can be subjective and inconsistent [26–29], potentially undermining student confidence [30, 31]. Digital systems, by contrast, tend to offer more objective and reproducible feedback [26, 27, 32–37]. However, some authors noted that purely digital systems may not validly assess all aspects of students’ work [26, 32]. Integrating digital and traditional feedback is therefore recommended to balance objectivity and pedagogical value [26, 33, 38–43].

Traditional feedback tools such as checklists or visual diagrams can be subjective [44–46]. Previous studies reported low interrater agreement among faculty [26], whereas the present study achieved an ICC > 0.827, indicating excellent reliability. Literature also shows that students often overestimate their own performance compared to teachers [45, 47, 48]. Including student self-evaluation and repeated blinded assessments in future research could provide valuable insights.

Digital assessment tools like prepCheck reduce subjectivity by comparing student preparations with calibrated master models, offering visualized deviations within defined tolerance levels [27, 45, 46]. Such systems can be adapted to training level and allow longitudinal performance tracking, enhancing feedback quality [45, 49]. Despite their advantages, implementation requires financial investment and sufficient training time for both staff and students [45, 46]. Once integrated, these systems can improve motivation, learning outcomes, and teaching efficiency [27, 45].

Regarding strengths of the present pilot study, a major strength is the highly standardized preclinical training environment, which enabled a controlled comparison between conventional visualization and microscope-assisted evaluation. In addition, outcomes were assessed using both analog and digital approaches, reflecting contemporary educational workflows in restorative dentistry. The use of structured preclinical scoring sheets contributed to transparent and reproducible outcome assessment. Importantly, the evaluation included raters with different levels of clinical training and experience (10th-semester dental student, dentist with 1 year of clinical experience, and senior clinician/attending dentist with 7 years of clinical experience). Furthermore, the use of generalized estimating equations (GEE) represents a methodological strength, as the presented approach accounts for correlated observations arising from repeated measurements. Overall, the present findings provide valuable feasibility data and hypothesis-generating evidence to support future confirmatory studies in larger cohorts and clinical settings.

Several limitations should be acknowledged. First, the present pilot study was conducted in a real undergraduate educational setting and included two cohorts at different stages of training (fifth and ninth semesters). Students from the fifth and ninth semesters were intentionally included to represent different training levels. However, differences in baseline manual dexterity and clinical experience between cohorts must be considered when interpreting results. Participants’ visual acuity was not formally assessed. Dental loupes were used with standard optical settings and without individualized corrective lenses. Potential uncorrected visual differences may therefore have influenced performance. While the present approach reflects the heterogeneity commonly encountered in preclinical teaching, it also limits direct comparability because baseline manual dexterity and prior operative experience may differ between cohorts and between individuals. Although randomization was performed within each cohort to reduce allocation bias, entering skill level could not be fully controlled and may have influenced preparation quality outcomes. Working time and participants´ age were not recorded as study variables in the present study and therefore no correlation analyses could be conducted. Evaluators with different levels of training and clinical experience were intentionally included to reflect the heterogeneity of real educational assessment environments. High ICC values indicate acceptable inter-rater reliability despite evaluator variability. Lower ICC values observed for digital assessments may reflect variability in interpretation of deviation maps rather than reduced measurement reliability.

Second, the study used a between-group design rather than a within-subject crossover design. A crossover approach in which the same participants sequentially perform preparations using SG, DL, and OPMI could better control for inter-individual variability in manual dexterity. Besides, a crossover design might better control for inter-individual variability; however, repeated preparations would introduce substantial learning effects. However, such a design was not feasible in our course setting and would likely introduce substantial learning and carryover effects from repeated preparations, making it difficult to isolate the effect of magnification from training-related improvement. Future studies should therefore consider longitudinal and/or crossover designs with repeated sessions and baseline skill assessments to more precisely determine the effect of magnification systems on preparation quality.

Although randomization was performed, the pilot sample size (*n* = 60) may have allowed residual imbalances in baseline skills and prior clinical experience between groups. Future confirmatory studies should consider stratified randomization (e.g., by semester level, baseline performance, or prior exposure to magnification devices) to ensure balanced group allocation.

Furthermore, the present study focused exclusively on an anterior tooth preparation, which is relatively accessible and well visualized under an operating microscope. The applicability and ergonomic advantages of magnification devices may differ more markedly in posterior regions, where limited mobility, restricted angulation, and indirect visualization play a greater role. Future studies should therefore compare anterior and posterior preparation tasks to evaluate whether dental loupes provide particular advantages in areas where microscope positioning may be more challenging.

An additional potential confounder is that clinical students may have already been familiar with the use of dental loupes from routine clinical training, potentially influencing performance independently of the experimental intervention. This prior exposure could partially explain the high performance observed in the DL group among clinical students, independent of the experimental visualization condition. Future studies should systematically record prior magnification experience and consider controlling for this factor in the analysis. Although structured scoring sheets were applied, certain criteria (e.g., polishing quality, margin contour) inherently involve subjective judgment. In general, several potential confounding factors must be considered. First, differences in training level between fifth- and ninth-semester students may have influenced preparation quality. Second, the between-group design does not fully control for inter-individual variability. Third, only anterior tooth preparations were evaluated. Fourth, participants’ visual acuity was not assessed. Finally, certain evaluation criteria inherently involve subjective judgment.

Key limitations of the present study include the pilot sample size, between-group design, cohort heterogeneity, absence of formal visual acuity testing, anterior-only task selection, potential prior magnification experience, and partial subjectivity of evaluation criteria.

Future investigations should concentrate on the use of an OPMI that might be associated with a distinct learning curve. Therefore, it is plausible that greater routine experience with OPMI could lead to even higher preparation quality compared with DL over time. Future longitudinal studies incorporating multiple training sessions are warranted to evaluate the long-term educational effect and whether increased familiarity with OPMI results in superior preparation outcomes. Repeated testing at regular intervals with larger, more heterogeneous cohorts (regarding experience, gender, and age) would allow robust conclusions. Expanding the number of raters per experience level and maintaining both digital and analogue evaluation methods are recommended to strengthen reliability and comparability.

## Conclusions

The present study was the first one to examine the use of different magnification devices on students performing a preparation and a provisional restoration. The use of visual magnification aids (OPMI and DL) were associated with improved performance regarding dental preparation and restoration procedures in dental education. While both tools proved superior to SG, their effectiveness varied depending on the students’ clinical experience and familiarity with the device. Dental microscopes were particularly advantageous for preclinical students whereas magnifying loupes yielded better results among clinical students.

The findings underscore the importance of structured training and adequate familiarization to optimize the benefits of magnification tools. Moreover, the combination of conventional and digital assessment methods provided the most consistent evaluation outcomes, emphasizing the need for standardized, objective grading systems. Integrating optical aids into dental curricula from the preclinical stage appears to be a valuable strategy for improving practical skills. Future longitudinal research with larger cohorts and expanded is needed to better understand the long-term impact of magnification devices on learning success, clinical performance, and student health.

## Data Availability

The datasets used and/or analysed during the current study are available from the corresponding author on reasonable request.

## Abbreviations

DL: Dental loupes
OPMI: dental operating microscope
SG: protective glasses
CAD/CAM: computer-aided design/computer-aided manufacturing
GEE: Generalized Estimating Equations
ICC: Intraclass Correlation Coefficient
